# Epigenetics of Most Aggressive Solid Tumors: Pathways, Targets and Treatments

**DOI:** 10.3390/cancers13133209

**Published:** 2021-06-27

**Authors:** Javier Martinez-Useros, Mario Martin-Galan, Maria Florez-Cespedes, Jesus Garcia-Foncillas

**Affiliations:** 1 Translational Oncology Division, OncoHealth Institute, Fundacion Jimenez Diaz University Hospital, Avenida Reyes Catolicos 2, 28040 Madrid, Spain; mariomgtics@gmail.com; 2 Imperial College London, Exhibition Road, South Kensington, London SW7 2BX, UK; maria.florez-cespedes17@imperial.ac.uk

**Keywords:** epigenetic, methylation, acetylation, non-coding RNA, small-cell lung cancer, triple-negative breast cancer, pancreatic ductal adenocarcinoma, glioblastoma, metastatic melanoma, advanced ovarian cancer

## Abstract

**Simple Summary:**

The large amount of knowledge regarding epigenetic pathways has opened a broad range of treatments that provide hope for adult patients with highly aggressive forms of solid tumors. The most commonly used treatments for epigenic modifications are based on the specific inhibitors of DNA methyltransferases, azacitidine and decitabine (5-AZA-dC), and on histone deacetylases inhibitors, such as trichostatin A (TSA) or vorinostat (SAHA). However, many other compounds are under investigation, and some are being evaluated in clinical trials. In this review, we have extracted relevant information about epigenetic pathways and treatments that target epigenetic modifications in highly aggressive tumors, as a new hope for these patients.

**Abstract:**

Highly aggressive tumors are characterized by a highly invasive phenotype, and they display chemoresistance. Furthermore, some of the tumors lack expression of biomarkers for target therapies. This is the case of small-cell lung cancer, triple-negative breast cancer, pancreatic ductal adenocarcinoma, glioblastoma, metastatic melanoma, and advanced ovarian cancer. Unfortunately, these patients show a low survival rate and most of the available drugs are ineffective. In this context, epigenetic modifications have emerged to provide the causes and potential treatments for such types of tumors. Methylation and hydroxymethylation of DNA, and histone modifications, are the most common targets of epigenetic therapy, to influence gene expression without altering the DNA sequence. These modifications could impact both oncogenes and tumor suppressor factors, which influence several molecular pathways such as epithelial-to-mesenchymal transition, WNT/β–catenin, PI3K–mTOR, MAPK, or mismatch repair machinery. However, epigenetic changes are inducible and reversible events that could be influenced by some environmental conditions, such as UV exposure, smoking habit, or diet. Changes in DNA methylation status and/or histone modification, such as acetylation, methylation or phosphorylation, among others, are the most important targets for epigenetic cancer therapy. Therefore, the present review aims to compile the basic information of epigenetic modifications, pathways and factors, and provide a rationale for the research and treatment of highly aggressive tumors with epigenetic drugs.

## 1. Introduction

DNA is organized inside the nucleus, in a very complex structure called chromatin. The negative charge of DNA is supported by basic proteins that are rich in arginine and lysine residues, called histones. There are five families of histones and according to their function they are called core histones (H2, H3, and H4) that form the nucleosome core, or linker histones (H1 and H5), which contribute to the condensation of the nucleosome. The nucleosome core is composed by two H2A–H2B dimers and a H3–H4 tetramer. The electrostatic attraction between the positively charged histones and negatively charged DNA allows the complex structure of chromatin to form [1,2]. Chromatin is composed of nucleosomes wrapped by 146–147 bp DNA [3]. The H1 histone serves as a linker between the nucleosomes, in order to provide a highly stable chromatin structure [4]. Histones possess amino-terminal tails that allow gene regulation, by epigenetic modifications, due to their flexible shaping [4]. Deregulation in the deposition of histone modification is associated with several human diseases, such as cancer [5]. Moreover, some epigenetic modifications could be influenced by specific molecular pathways involved in cancer, such as epithelial-to-mesenchymal transition (EMT) [6], Wnt/β-catenin signaling [7], the MAPK signaling pathway [8], DNA repair [9], hypoxia [10], and the PI3K–mTOR pathway [11]. Interestingly, some environmental conditions, such as UV exposure or diet, are also able to induce epigenetic changes. For example, compounds such as folate, choline, betaine, and methionine act as cofactors or methyl donors for DNA methylation reactions. A diet rich in resveratrol, curcumin, genistein, epigallocatechin-3-gallate, sulforaphane, and quercetin is able to reactivate certain tumor suppressive genes by inducing DNA demethylation; however, fungi-contaminated agricultural foods contain mycotoxins that may also lead to cancer [12].

Clinical research has achieved several advances in cancer treatment that have led to a longer survival of patients. However, treatment strategies for highly aggressive tumors remains almost constant, without any significant improvements. In the new era of targeted therapy, epigenetic therapies appear as a potential approach for the treatment of highly aggressive tumors, offering new hope for these patients. Methylation and hydroxymethylation of DNA, and histone modifications, are the most common targets of epigenetic therapy, to influence gene expression without any DNA alteration. On the other hand, increasing reports support the use of non-coding RNA as epigenetic treatment to intercept translation, and negatively regulate the expression of oncogenes.

### 1.1. DNA Methylation

**Figure 1:**
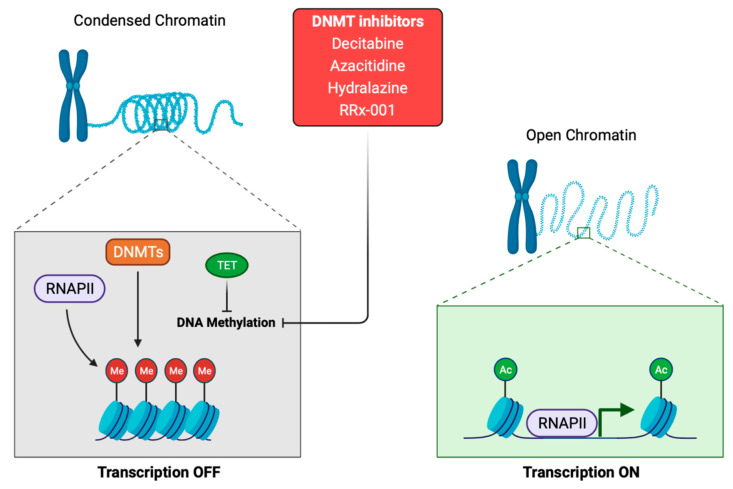
Schematic representation of the DNA methylation process. DNMTis inhibits DNA methylation by downregulation of DNMTs. The action of DNA methylation inhibitors (DNMTs inhibitors and TET proteins) triggers a chromatin-remodeling process and chromatin structure becomes transcriptionally accessible to RNA polymerase II, which will begin the transcription process. DNMTis: DNA methyltransferases inhibitors. DNMTs: DNA methyltransferases. TET: ten-eleven translocation proteins. RNAPII: RNA polymerase II. Me: methyl. Ac: acetyl.

DNA methylation plays a crucial role in normal cell metabolism; therefore, changes in the methylation status of cells, by methyltransferases, can lead to cell transformation and represent the difference between normal and tumor cells [13] (Figure 1). Cytosine and adenine are the only bases susceptible to methylation. DNA methylation consists of the transfer of methyl groups (-CH_3_) to the cytosine in position C5, which is followed by a guanine (G). These sites are termed CpG dinucleotides and result in 5-methylcytosine. These sites occur with high frequency in CpG genomic regions. Non-cytosine methylation, such as the methylation of adenine or thymine, appears in very low probability [14]. CpG islands are located in ~60% of human promoters, and methylation of these sites results in a transcriptional repression of the genes [5,15]. Furthermore, 60–80% of CpG islands of somatic cells genome are methylated [16]. The DNA methyltransferase (DNMT) (Figure 1) family regulates the process of DNA methylation [17]. This protein family is composed of the following five members: DNMT1, DNMT2, DNMT3a, DNMT3b and DNMT3L. Interestingly, mutations in some of these members are usually associated with some types of cancer [18]. For example, the DNMT3b subtype is significantly overexpressed in some tumors [19,20]. The methylation status of DNA can be read by MBD (methyl-CpG binding domain) proteins, which are divided into three families. The first family includes MeCP2, MBD1, MBD2, MBD3 and MBD4 [21]; although, MBD3 can only detect hydroxymethylated DNA [22]. The second family is characterized by a BTB domain (also called as the POZ domain) and comprises ZBTB33, ZBTB4 and ZBTB38 [23]. The third family includes the following two proteins: UHRF1 and UHRF2 [24]. Some drugs are able to modulate the expression levels of these proteins. Decitabine and 5-azacytidine trigger calcium-calmodulin kinase (CamK) activity, leading to MeCP2 nuclear export, which induces the epigenetic reactivation of some tumor suppressive genes in colorectal cancer [25]. Other drugs, such as 5-azacytidine, doxorubicin, vorinostat, paclitaxel, or cisplatin, regulate the expression of different MBD proteins. MBD1 was upregulated after treatment with all those drugs. Downregulation of MBD2 was observed after 5-azacytidine, doxorubicin, or vorinostat treatment, MBD3 downregulation after vorinostat, and the inhibition of MBD4 varied in a time- and drug-dependent manner [26]. Another study reported the decrease in ZBTB4 levels after roscovitine treatment [27]. Concerning UHRF1, its downregulation enables the demethylation, and the subsequent reactivation, of some epigenetically silenced tumor-suppressive genes [28]. Giovinazzo et al. reported the pharmacological inhibition of UHRF1 by the anthracycline derivatives, idarubicin and mitoxantrone [29]. Therefore, several drugs allow the negative modulation of these MBD proteins, implying a high potential to be used as target therapies.

Aberrant DNA methylation has been associated with drug resistance, and as predictive biomarker [30]. Also, inadequate methylation is associated to inflammatory diseases, pre-malignant lesions and cancer led by chromatin instability [31]. Hypermethylation and hypomethylation of DNA are usual phenomena in cancer; indeed, tumor-suppressive genes are hypermethylated in cancer cells, while they remain hypomethylated in normal cells [32]. Therefore, the demethylation of target genes could be a promising approach in clinical practice. Physiologically, demethylation of DNA sequences is carried out by the ten-eleven translocation (TET) proteins. The three mammalian TET proteins, called TET1, TET2 and TET3, enable the oxidation of 5-methylcytosine (5mC) of nucleic acids, to 5-hydroxymethylcytosine (5hmC), 5-formylcytosine (5fC) or 5-carboxylcytosine (5caC) [33]. The mutation or inhibition of TET proteins is associated with aging and tumorigenesis [34]. Indeed, mutation in *TET2* is frequently found in hematopoietic malignancies [35], and the downregulation of TET proteins has been observed in several solid tumors, such as breast cancer, gastric, glioblastoma, liver, lung, melanoma and prostate [34,36,37,38].

### 1.2. Histone Modification

**Figure 2:**
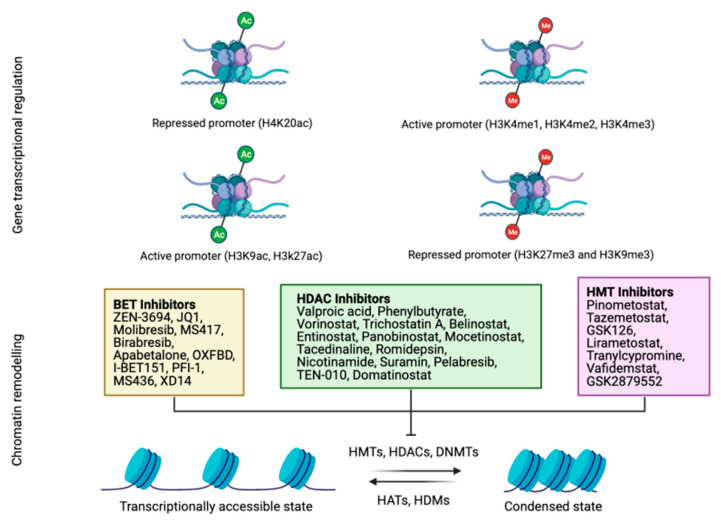
Schematic representation of the main histone modification processes. Both acetylation and methylation positively and negatively regulate gene transcription according to the methylated or acetylated residue (up). Several drugs have been designed to allow chromatin remodeling by the inhibition of BET, HDAC or HMT proteins that condense chromatin and hamper transcription (down). Histones acetylation and cytosines unmethylation will result in an open chromatin structure and gene transcription is active. BET: bromodomain and extra-terminal motif (BET) proteins. HDAC: histone deacetylases. HMT: histone methyltransferase. DNMTs: DNA methyltransferases. HAT: histone acetyltransferase. HDM: histone demethylase.

Histone modification can take place in the following two locations: the flexible tails of the nucleosomes and the internal sites in the core of the histone (Figure 2) [39]. The residues most susceptible for modification are lysine and arginine residues, and hydroxyl group-containing serine/threonine/tyrosine residues [40]. Histone modification includes several reactions, such as the methylation and acetylation of lysine and arginine residues, phosphorylation of threonine and serine residues, SUMOylation of lysine residues, isomerization of proline residues, ADP-ribosylation, ubiquitylation, citrullination, deamination, formylation, O-GlcNAcylation, propionylation, butyrylation and crotonylation [41]. Histone acetylation of lysine limits the interactions between the histones H3 and H4, and DNA; while deacetylation leads to gene inactivation [42]. Acetylation is associated with active transcription, and facilitates the recruitment of co-regulators and elements to promote transcription. Modifications of histones are driven by protein effectors and are crucial in the regulation of gene expression. HATs (histone acetyltransferases) are a group of effectors that transfer the acetyl groups to lysine residues of histones [43]. Notably, aberrations in the histone modification pattern may induce cancer [44]. For example, tumor cells present a loss of Lys16 acetylation and Lys20 trimethylation of histone H4 at the early phase of tumor initiation [45]. In contrast, histone deacetylases are another group of effectors that remove the acetyl groups from acetyl-lysine residues, which allows DNA to wrap tightly to histones [46]. Histone deacetylases (HDACs) have been recently reported as a target for cancer therapy (Figure 2) [46]. HDAC1-11 and other histone deacetylases, termed sirtuins, normally play a role as gene silencers [47]. Other effectors are histone demethylases that remove methyl groups from lysine residues. The lysine-specific demethylase 1 (LSD1) exhibits tumor-prone abilities in glioblastoma, and its inhibition sensitizes tumor cells to vorinostat, increasing apoptosis [48]. Other histone demethylases, such as KDM4, produce genome instability, while KDM6 is considered a tumor-suppressive factor [49].

On the other hand, readers of these modifications determine the functional outcome of specific epigenetic change. Some of the proteins involved in the recognition of histone modifications are BET (bromodomain and extraterminal domain-containing). This family is composed of four proteins (BRD2, BRD3, BRD4 and BRDT), and plays important roles in tumor development, since they also lead to transcriptional activity [50,51]. For this reason, BET inhibitors have been evaluated as anti-tumor therapies, showing encouraging results in several malignancies, without significant toxicities or adverse events (Figure 2) [51].

### 1.3. Non-Coding RNA

**Figure 3:**
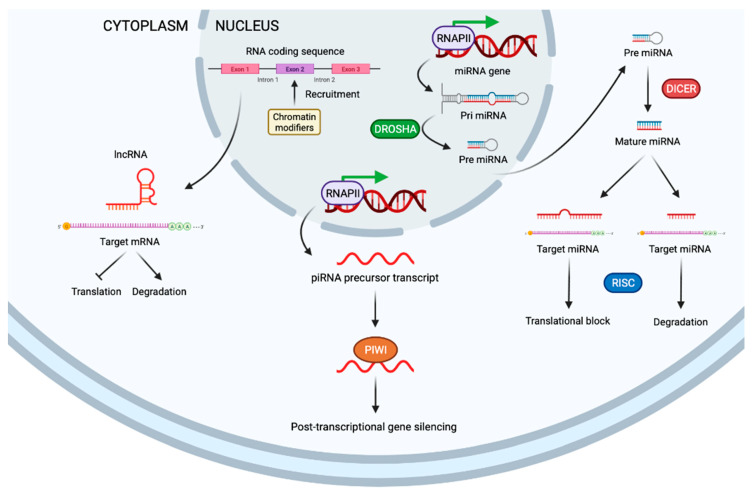
Schematic representation of non-coding RNA. LncRNA targets mRNA to inhibit translation or degrade mRNA (left). PIWI proteins stabilize piRNAs and lead to post-translational control (middle). MiRNA are originated from double-stranded RNA hairpins. The ribonuclease III enzyme, DROSHA, binds and cleaves hairpin structures in primary RNA transcripts into precursor miRNAs. Once transported to cytoplasm, precursor miRNAs are processed by DICER into mature miRNAs that regulate expression of mRNA (right). miRNA: microRNA. DICER: ribonuclease III enzyme. DROSHA: ribonuclease III enzyme. RISC: RNA-induced silencing complex. piRNA: PIWI-interacting RNA.

This family includes several factors, but the most notable, in regards to cancer, are small interfering RNA (siRNAs), microRNAs (miRNAs), PIWI-interacting RNA (piRNAs), and long non-coding RNAs (lncRNAs) (Figure 3) [5].

The small interfering RNA (siRNA) transcripts are double-stranded RNA fragments, about 21–25 base pairs long. The function of siRNA is thought to be related to erasing viral double-stranded sequences to avoid infection. SiRNA is cleaved by Dicer from long double-stranded RNA sequences [52]. The double-stranded siRNA is processed by the RNA-induced silencing complex (RISC), to produce single-stranded siRNA [53]. This strand is able to recognize the target mRNA. The perfect match induces mRNA degradation, and a partial match results in translational repression [54].

MiRNA are the most known non-coding RNA and they are involved in several cell functions. Several miRNAs are linked to cancer initiation and development. Furthermore, miRNAs can be tumor-prone or tumor-suppressive factors [55]. MiRNAs are very similar to siRNAs; however, miRNAs originate from double-stranded RNA hairpins, rather than long double-stranded RNA that need additional manipulation by DROSHA [56].

P-element-induced wimpy testis (PIWI) proteins belong to the Argonaute (AGO) family and were discovered in the germline [57]. They also bind a unique type of non-coding small RNAs, called piRNAs (PIWI-interacting RNAs). This tandem, composed of PIWI and piRNAs, constitute the piRNA-induced silencing complex (piRISC). PiRNAs are special mediators, because depending on the factors that modulate, some piRNAs are considered oncogenic, while others are considered tumor-suppressive factors [58].

Long non-coding RNAs (lncRNAs) constitute a huge subgroup of ncRNAs, defined as RNA transcripts, with more than 200 nucleotides [59]. LncRNAs play an important role in the development of various cancers [60]. The lncRNA, HOTAIR, is closely related to epigenetic modifications. The knockdown of HOTAIR activates transcription-reducing H3K27 trimethylation [61]. Moreover, HOTAIR is able to interact with lysine-specific histone demethylase 1A (LSD1) [62]. Aberrant HOTAIR expression has been observed in several tumors, and its positive expression has been associated with several hallmarks of cancer, such as high cell proliferation, angiogenesis or drug resistance, by the direct regulation of several downstream factors involving multiple signaling pathways [63,64,65]. Another crucial lncRNA is MALAT-1 (metastasis-associated lung adenocarcinoma transcript-1), which is aberrantly upregulated in multiple tumor types, and yields high proliferative and metastatic profiles [66]. High expression of MALAT-1 has been associated with high-grade and advanced-stage melanoma, glioma and lung cancers [67,68,69].

The large amount of knowledge regarding epigenetic modifications has opened a broad palette of treatment strategies for the most aggressive solid tumors in adulthood. Thus, the objective of this review is to compile basic knowledge about epigenetic pathways and treatments, and provide a rational for further clinical trials, based on the use of these treatments in highly aggressive solid tumors.

## 2. Epigenetic Modulation in Highly Aggressive Solid Tumors

**Figure 4:**
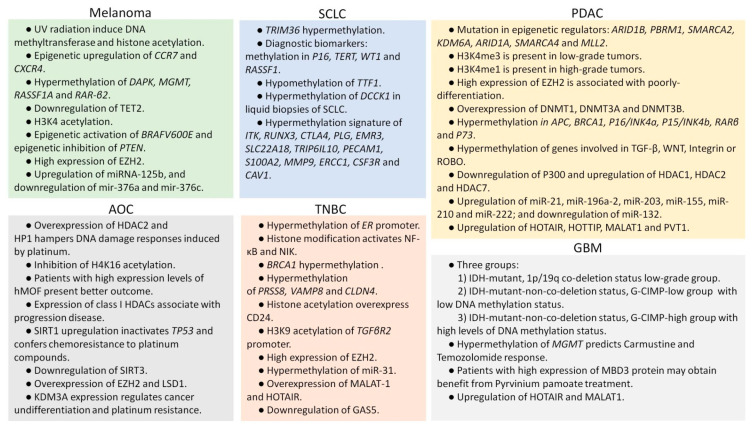
Summary of the most representative epigenetic modifications of most aggressive solid tumors observed in adulthood. UV: ultraviolet. SCLC: small-cell lung cancer. PDAC: pancreatic ductal adenocarcinoma. AOC: advanced ovarian cancer. TNBC: triple-negative breast cancer. GBM: glioblastoma.

The most commonly drugs used as hypomethylating agents are specific inhibitors of DNA methyltransferase (DNMT), for example, azacitidine and decitabine (5-AZA-dC) (Figure 1) [70]. These drugs lead to a reduction in whole DNA methylation status [71], and damage DNA by inducing genomic instability that hampers DNA synthesis [72]. Trichostatin A (TSA) and vorinostat (SAHA) are the most used inhibitors for the class I and II histone deacetylases (HDAC), demonstrating a broad spectrum of epigenetic activities [73]. Sodium phenylbutyrate is also a histone deacetylase inhibitor that is under investigation for its potential use in malignant brain tumors [74]. Although epigenetic drugs have a great potential to improve patient prognosis, there are also important considerations concerning global transcriptional effects. Epigenetic modifications by drugs may result in an aberrant gene expression pattern, leading to a global transcriptional alteration that will drive severe genome instability and cancer [75]. At the molecular level, several studies have reported the upregulation of P21 after epigenetic treatment [76]. On the other hand, since germ cells drive broad epigenetic reprogramming, these drugs could influence histone modifications and alterations in the non-coding RNAs of sperm and oocytes, which may influence progeny development [77]. Furthermore, these drugs have been demonstrated to impair normal hematopoiesis. Indeed, some of the adverse events in the clinical evaluation of epigenetic drugs are hematologic toxicity [76], as well as severe cardiac toxicity, as previously reported with the administration of the histone deacetylase inhibitor [78]. Nevertheless, these drugs exhibited promising results for cancer patients, and due to the growing interest and hope in epigenetic modulation in the clinical practice, we focus this review on different pathways and treatments for the most aggressive solid tumors, specifically small-cell lung cancer, triple-negative breast cancer, pancreatic ductal adenocarcinoma, glioblastoma, metastasic melanoma and ovarian cancer (Figure 4).

### 2.1. Epigenetic Modulation in Small-Cell Lung Cancer

Small-cell lung cancer (SCLC) incidence over time has decreased, reducing by 10–11% in all the cases of lung cancer, which may reflect decreases in smoking habits and changes in the type of cigarettes [79]. One of the causes that leads to a malignant phenotype in lung cancer is the exposure to polycyclic aromatic hydrocarbons, such as benzo (a) pyrene. This induces *TRIM36* hypermethylation, and its subsequent inhibition is associated with the acquisition of an aggressive phenotype [80]. SCLC is the highest aggressive subtype of lung cancer, since tumor cells are highly proliferative, and they spread and metastasize quickly throughout the body [81].

The methylation status of bronchial washings from different types of lung cancers provided a signature, based on four DNA methylated factors (*P16*, *TERT*, *WT1*, and *RASSF1*), which could improve the efficiency of SCLC diagnosis when compared with cytologic evaluation [82]. Another study found that SCLC frequently express thyroid transcription factor 1 (TTF1) at high levels, due to hypomethylation of its promoter [83]. TTF1 overexpression has been reported to confer high tumor cell proliferation and survival [84]. Also, the hypermethylation status in *DCLK1*, which has been associated to colorectal cancer and cholangiocarcinoma, has been found in liquid biopsies in 75% of SCLC patients, and has been associated with poor survival; therefore, this could represent a promising biomarker for early diagnosis and disease prognostic for this cancer subtype [85]. Several other genes have also been found methylated in SCLC, for example *ITK*, *RUNX3*, *CTLA4*, *PLG*, *EMR3*, *SLC22A18*, *TRIP6IL10*, *PECAM1*, *S100A2*, *MMP9*, *ERCC1*, *CSF3R* and *CAV1* [86].

In the treatment scenario, one study reported that 5-AZA-dC and the HDAC inhibitors, LBH589 or MGCD0103, synergistically reduced proliferation in five out of nine SCLC cell lines in vitro [87]. Interestingly, the authors observed higher expression of IFN-stimulated genes in the resistant cell lines after treatment, which determine SCLC cell sensitivity to epigenetic modulators [87]. Another study describes that TSA is able to induce an increase in ABCB1, a protein that confers drug resistance to tumor cells [88]. In clinical trials, a new epigenetic treatment, called RRx-001, is under investigation (NCT02489903; Table 1; Figure 1 and Figure 2). RRx-001 is an alkylating agent based on a dinitroazetidine derivative that inhibits DNA methyltransferase (DNMT) and induces DNA damage via ATM/γ-H2AX, and apoptosis by the activation of caspases [89]. This drug is being tested in platinum refractory or resistant SCLC patients, with 3.8% complete responses and 23.1% partial responses, which increased the overall survival OS [90].

The progress in the treatment of SCLC has been very limited in the last decade, especially when compared to the numerous results that arise for NSCLC. Although the FDA approved the use of immunotherapy anti-PD-L1 in combination with carboplatin and etoposide as an induction therapy in extensive-stage SCLC, much remains to be done to achieve a cure for SCLC patients. In fact, the combination of immunotherapy plus chemotherapy has only represented an improvement in the overall survival of two months [91]. Therefore, there is much left to be done, and, in this sense, drugs directed against epigenetic targets may represent potential treatment approaches.

### 2.2. Epigenetic Modulation in Triple-Negative Breast Cancer

Triple-negative breast cancers (TNBC) comprise 7–14% of all breast cancers [92]. TNBC is considered the most aggressive subtype due to the lack of expression of estrogen receptors (ER), progesterone (PR), and HER2 receptors that make the currently used drugs ineffective. One study reported a highly methylated promoter region in the ER gene [93]; thus, a correlation has been suggested with the downregulation of ER expression levels in TNBC patients and the absence of a response [94]. Histone H3 methylation and deacetylation lead to a less compact chromatin structure, which facilitates DNA access to transcription protein machineries. For example, one of the activated genes, due to histone modification in TNBC that provides proliferative features, is NF-κB and its NF-κB-inducing kinase (NIK) [95].

A high percentage of TNBC patients carry germline/somatic mutations or epigenetic silencing in *BRCA1*, which implies a deficient DNA repair machinery. Genome-wide DNA methylation analysis in TNBC supports that hypermethylation causes the downregulation of PRSS8, VAMP8 and CLDN4 factors, which confer mesenchymal features [96]. One study revealed a high incidence of *BRCA1* methylation in a TNBC basal-like subtype. This finding could imply resistance to PARP inhibitors for the treatment of *BRCA*-mutant basal-like TNBC [97]. As most of the cases carry mutations in *TP53*, one study has demonstrated that the use of zinc metallochaperones (ZMCs) is efficient to reactivate zinc-deficient mutant *TP53*, by restoring its zinc binding. The use of ZMC1 with a mutation in *TP53^R175H^* restores *TP53* reactivation [98]. Another mechanism altered by epigenetic modifications in TNBC is the epithelial-to-mesenchymal transition (EMT). The combination of the methyltransferase inhibitor, SGI-110, with the histone deacetylase inhibitor, MS275, has shown a high antitumor ability against TNBC, by epigenetically targeting EMT. Here, TNBC cells showed a marked upregulation of the epithelial protein E-cadherin, and WNT inhibition, and reduced nuclear translocation of EpCAM, which reversed the mesenchymal phenotype after treatment [99]. CD24 overexpression is associated with histone acetylation and is an independent poor prognostic factor in TNBC; importantly, CD24 may be a potential therapeutic target for this type of breast cancer [100]. Mutation analysis revealed that a novel carbazole, SH-I-14, disrupted the STAT3 –DNMT1 interaction and led to the re-expression of tumor-suppressive genes such *PDLIM4* or *VHL*, through demethylation, and showed a high anti-proliferative effect in TNBC models [100].

Concerning histone acetylation, one study showed high levels of H3K9 acetylation in the *TGFβR2* promoter in the TNBC cell line, MDA-MB-231. Moreover, the inhibition of *TGFβR2* decreased migration of the cell line [101]. Another factor, the enhancer of zeste homolog 2 (EZH2), is a type of histone methyltransferase that is highly expressed in TNBCs, and its expression implies shorter disease-free survival in TNBC patients [102]. EZH2 works together with HDACs to mediate transcription repression, by increasing histone H3 Lys27 trimethylation (H3K27me3). One study reported that the inhibition of EZH2 increases H3 Lys27 acetylation, which promotes open chromatin transcription activation, and induces apoptosis in TNBC, through the upregulation of B-cell lymphoma-2-like 11 (BIM) [103].

In respect to ncRNA, the presence of hypermethylation at miR-31 loci in TNBC has been described. Moreover, miR-31 maps to the sequence of a novel long non-coding RNA, LAOT554202 [104]. Both are downregulated in TNBC; however, epigenetic treatment was shown to increase both miR-31 and LAOT554202 expression [104].

Also, the deregulation of some lncRNAs has been associated with the progression of different breast tumors [105]. It has been described that high levels of MALAT1 have correlated with tumor aggressiveness and poor survival of TNBC patients [106,107]. Another lncRNA, HOTAIR, is commonly upregulated in TNBC and associated with the invasive phenotype [108] and lymph node metastasis [109]. In contrast, GAS5 has a protective effect against TNBC, and its overexpression suppressed tumor progression [110], and increased sensitivity to paclitaxel and the subsequent apoptosis ratio [111]. A meta-analysis from 21 studies reported that patients with upregulation of HOTAIR and MALAT1, among others, and downregulation of GAS5 and another three lncRNAs, presented poor survival rates [112]. Another meta-analysis supported that the expression of some lncRNAs, such as MALAT1 and HOTAIR, are associated with positive lymph nodes, while the expression of GAS5 exhibited the opposite effect [113]. Although the FDA has approved epigenetic agents to overcome chemoresistance, to reverse DNA methylation (e.g., 5-azacytidine), and to reverse histone deacetylation (e.g., Trischostatin A and vorinostat (SAHA)), the efficacy of 5-azacytidine has not been consistent in breast cancers. Currently, a new BET inhibitor, ZEN-3694, is being tested in clinical trials because of its ability to prevent the interaction between the BET proteins and acetylated histones (Figure 2). ZEN-3694 is being evaluated in TNBC patients without germline mutations of *BRCA1* or *BRCA2* (NCT03901469; Table 1). Another phase I/II clinical trial is based on the reactivation of ER by decitabine and the histone deacetylase inhibitor, LBH589, in order to enhance the subsequent tamoxifen treatment (NCT01194908; Table 1).

Modification of the epigenetic machinery is a new tool for the treatment of TNBC, especially BET inhibitors. These drugs have already shown positive effects in preclinical models, and they have yet to be evaluated in clinical trials. These new drugs against epigenetic targets have the potential to decrease tumor aggressiveness and increase sensitivity to standard treatments. Maybe in the foreseeable future, these treatments will improve patient prognosis.

### 2.3. Epigenetic Modulation in Pancreatic Ductal Adenocarcinoma

Pancreatic ductal adenocarcinoma (PDAC) shows the lowest five-year survival rate, around 3%, and it is the fourth leading cause of cancer-related deaths in men and women [114]. It is often misdiagnosed and the symptoms are commonly treated by ambulatory care, leading to a late diagnosis; thus, patients present metastatic disease in ~80% of cases at diagnosis. Furthermore, it exhibits chemoresistance due to a complex link between the tumor cells and their microenvironment [115]. In PDAC, most of the studies are centered on mutations in *SMAD4*, *TP53*, *KRAS* or *CDKN2A*, which happen in more than 50% of patients [116]. Furthermore, the mutation in *MBD4* has been found in PDAC, with microsatellite instability [117]. A recent study discovered mutations and genetic variants in several epigenetic regulators, such as *ARID1B*, *PBRM1*, *SMARCA2*, *KDM6A*, *ARID1A*, *SMARCA4*, and *MLL2* [118]. In addition, PDAC has a broad epigenetic signature, which activates oncogenes and inactivates tumor-suppressive genes [119]. Both high- and low-grade PDAC exhibit specific epigenetic features associated with gene expression patterns. In low-grade PDAC, a highly enhanced H3K4me3 domain has been found, while in high-grade PDAC, a higher H3K4me1 signal was found [120]. Increased expression of DNMT1, DNMT3A and DNMT3B has been detected in PDAC, which suggests direct involvement in the epigenetic regulation of tumor progression [121]. In fact, hypermethylation has been found in *APC* (47.9% of cases), *BRCA1* (45.8%), P16/*INK4a* (35.4%), P15*/INK4b* (35.4%), *RARβ* (35.4%), and *P73* promoters (33.3%) in PDAC patients. Moreover, other genes are methylated to impair several signaling pathways, such as TGF-β, WNT, integrin or ROBO [122].

Concerning histone-modifying enzymes, aberrant HATs and HDACs have been found in PDAC. One study, performed in PDAC-derived cell lines, showed an inhibition of the expression of HAT, P300, and a secondary upregulation of several miRNAs [123]. The supplementary missense mutation in *P300* supports its role as a tumor-suppressive gene in PDAC [124]. The aberrant expression of HDACs is frequently observed in PDACs. For example, HDAC2 and HDAC7 expressions are increased in PDACs, especially in poorly differentiated cases [125,126]. Also, the overexpression of HDAC7 clearly differentiates PDAC from other benign pancreatic neoplasms. A study found that HDAC1 was overexpressed in 56% of PDAC and PanIN lesions [127]. Other studies suggest that RNF2 allows ubiquitination of H2A and downregulation of RNF2, which inhibits tumor proliferation in PDAC in vitro [128]. Histone acetyltransferase (HAT) inhibitors impact genome-wide H3K27ac patterns of PDAC cells [120]. The HAT inhibitors ICG-001 and C646 also impair gene expression and inhibit tumor growth in PDAC [129].

Concerning miRNA, one study with PDAC patients revealed a poor prognosis signature based on the deregulation of 64 miRNAs, and the upregulation of miR-21, miR-196a-2, miR-203, miR-155, miR-210, and miR-222 [130]. Further studies confirmed a decreased expression of miR-132 in PDAC by promoter methylation [131]. Also, lncRNAs have appeared as important regulators for PDAC tumorigenesis [132]. HOTAIR, HOTTIP, MALAT1, and PVT1 are the most studied oncogenic lncRNA in PDAC [133], while LINC00673 and H19 are potential tumor suppressors [134,135]. PIWI-interacting RNAs (piRNAs) and their association with the PIWI subfamily of Argonaute proteins are crucial in pancreatic cancer progression. Indeed, PIWIL1 and PIWIL2 proteins are downregulated in PDAC, probably due to CpG island methylation [136].

The impact of bromodomain inhibitors has also been evaluated in PDAC. BRD4770 is an inhibitor of G9a that induces PDAC autophagy [137]. Moreover, histone methylation regulatory genes, such as KDM6A, are expressed and considered a new candidate in PDAC tumorigenesis [118]. KDM6A is an H3K27me3 demethylase, which is necessary for endoderm differentiation [138]. Another study reported that regions with loss of KDM6A sensitize PDAC cells to bromodomain inhibitors [139]. Other factors have been involved in the progression of PDAC. For example, EZH2 is an H3K27 methyltransferase that has been shown to be overexpressed in PDAC cell lines and patients [140]. The high expression of EZH2 is associated with an aggressive, poorly differentiated subgroup, which shows a shorter survival of patients [141]. Treatments based on the EZH2 inhibitor, DZNep, enhanced the effect of gemcitabine in tumor-derived cell lines and primary cultures from PDAC [142]. Small-molecule inhibitors against EZH2, which are currently being investigated as target therapies against PDAC, are as follows: EPZ-6438, GSK126, CPI-169 and UNC-1999 [143]. High expression of KDM2B is found in PDAC, and it associates with *KRAS^G12D^* to promote tumor initiation in in vivo models [144]. It has been reported that histone H3 modification of the *MUC2* promoter region regulates *MUC2* gene expression, and this expression could be positively modulated by treatment with trichostatin A (TSA) and 5-aza [145]. Another significant treatment is based on the inhibition of telomerase, through the following epigenetic mechanism: methyl-2-cyano-3,12-dioxooleana-1,9(11)-dien-28-oate (CDDO-Me). This drug is able to decrease cell proliferation and induce apoptosis in PDAC, through the inhibition of the DNA methyl transferases DNMT1 and DNMT3a [146]. Another strategy with 5-aza-dC in combination with a MEK inhibitor is able to induce cell cycle arrest [147]. Interleukin-13 receptor α2 (IL-13Rα2) is a tumor-associated antigen and a potential target for cancer therapy. Indeed, histones at the IL-13Rα2 promoter region are highly- acetylated; thus, treatment with HDAC inhibitors enhanced the expression of IL-13Rα2 and allowed sensitization for a second treatment [148].

In clinical trials, a pilot study with relapsed patients (NCT02847000; Table 1) tested decitabine in combination with tetrahydrouridine, a cytidine deaminase inhibitor, to avoid catabolism of decitabine. In this study, investigators found scarce effect, due to the local and systemic overexpression of cytidine deaminase in metastatic patients; the resectable patients did not overexpress this protein. This suggested a need for even higher tetrahydrouridine doses in advanced stages [149]. Another phase II trial with resectable PDAC is ongoing, to improve survival with oral azacitidine (CC-486); it includes high-risk patients that have positive lymph nodes, positive margins and/or elevated CA19-9 levels (NCT01845805; Table 1; Figure 1). In another study, with advanced or metastatic PDAC patients, only the patients treated with the combination of azacitidine plus nab-paclitaxel completed the treatment [150]. Previously, other studies have set the bases for the use of romidepsin with small-molecule inhibitors, to target both the MAPK and PI3K signaling pathways to increase apoptosis in RAS-mutated tumors, such as PDAC [151]. Currently, a new clinical trial against PDAC is active, to determine the safety and tolerability of azacitidine and/or romidepsin, combined with nab-paclitaxel/gemcitabine, followed by anti-PD-L1 and lenalidomide (NCT04257448; Table 1). Despite the vast epigenetic landscape of PDAC, clinical and translational research is opening broad treatment perspectives with hopeful results, which involve modulation of the immune response, or administration of epigenetic therapies alone or in combination with standard chemotherapy, to improve patients survival.

### 2.4. Epigenetic Modulation in Glioblastoma

Glioblastoma (GBM) is the most commonly diagnosed tumor in elderly Caucasian men [152]. Unfortunately, there is no effective treatment for GBM and the standard treatment for such brain tumors comprises surgical resection with concomitant chemoradiotherapy with temozolomide, followed by adjuvant chemotherapy [153]. However, the main handicaps achieving a successful recovery are tumor heterogeneity, chemoresistance of cancer stem cells, and diffusion of drugs through the blood–brain barrier. Based on molecular profiling, GBMs are classified into the following three major groups: (1) the 1p/19q co-deletion status group, consisting of the IDH-mutant-1p/19q co-deletion status low-grade group; (2) the G-CIMP-low group, including IDH-mutant non-co-deletion status with low DNA methylation status; and (3) the G-CIMP-high group, including the IDH-mutant non-co-deletion group with higher global levels of DNA methylation. IDH mutants lead to major epigenetic changes, because they produce the onco-metabolite 2-hydroxyglutarate that hampers iron-dependent hydroxylases, which includes the 5′-methylcytosine hydroxylases belonging to the TET family [154]. Among these, the second group, G-CIMP-low, has the worst prognosis [155].

*MGMT* (O-6-methylguanine DNA methyltransferase) hypermethylation predicts BCNU (carmustine) and temozolomide response in gliomas [156,157]. Moreover, patients with hypermethylation of MGMT showed longer overall survival than patients without methylation (43 vs. 16 months, respectively), and a longer time to progress (36 vs. 11 months, respectively) [158]. Treatment with temozolomide combined with the HDAC inhibitor suberoylanilide hydroxamic acid (SAHA) delayed temozolomide resistance when compared with treatment with temozolomide alone, by MGMT overexpression [159]. Some HDAC inhibitory prodrugs of butyric acid and valproic acid increased the antitumor efficacy of doxorubicin, without cardiotoxicity, in mouse models of GBM (Figure 2) [160].

Recently, it has been described that a specific GBM subtype, with high levels of MGMT, expresses methyl-CpG binding domain 3 (MBD3) protein, which targets CK1A. Therefore, this subtype of patients may obtain benefit from CK1A activator pyrvinium pamoate (Pyr-Pam), leading to MBD3 degradation [161]. The new histone deacetylase inhibitor CKD5 is a derivative of 7-ureido-N-hydroxyheptanamide, and it revealed strong antitumor effects in GBM, both in in vitro and in vivo models. The use of the demethylases KDM1 and KDM5A was also evaluated as a potential therapeutic target [161]. A study demonstrated that the inhibition of KDM1 and KDM5A showed a significant antitumor effect in wild-type and temozolomide-resistant GBM cells [162]. Another study tested the multi-KDM inhibitor JIB-04, which has strong anti-clonogenic activity in wild-type and temozolomide-resistant GBM cell lines [163]. Another potent HDAC6 inhibitor, CAY-10603, is able to induce apoptosis in several GBM primary and stem cell-like cell lines [164]. Another study, with small molecules such as EZH2 and HDACi, achieved proliferation arrest of GBM [165]. Treatment with vorinostat (HDAC inhibitor) and tranylcypromine (histone lysine demethylase KDM1A inhibitor) (Figure 2) decreased GBM stem cell proliferation and led to significant tumor regression in mouse models [166]. Also, the use of bromodomain inhibitors have risen in popularity, due to enhanced tumor lethality [167]. In fact, the BET inhibitor caused downregulation of the lncRNA HOTAIR, which induced cell cycle arrest in GBM cells [168]. Several signaling pathways, such as WNT/β-catenin, mTOR, or P53-HIF, are found to be activated in gliomas, due to the downregulation of several lncRNAs [63]. The inhibition of HOTAIR leads to the increased expression of miR-326, which induces the expression of FGF-1 [169]. Another lncRNA, MALAT1, which is upregulated in temozolomide-resistant GBM, has been seen to promote miR-101, miR-203 and thymidylate synthase expression when downregulated [170,171].

Concerning clinical trials, the use of temsirolimus has obtained interesting improvement in 36% of treated patients; furthermore, the treatment achieved a significantly longer time to progress [172]. In contrast, panobinostat administration with bevacizumab did not show any significant improvement in progression-free survival compared to bevacizumab alone [173]. A phase I/II trial with a histone deacetylase inhibitor, romidepsin, found this drug to be inefficient for patients with recurrent GBM [174]. Currently, a phase I clinical trial is ongoing, to test whether folic acid is able to lead to MGMT methylation and improve temozolomide plus radiation treatment in grade IV tumors (NCT01700569; Table 1). This trial was based on the fact that folate could induce DNA methylation and increase the sensitivity to temozolomide in in vivo models [175].

In conclusion, although molecular diagnosis has brought new options to identify and treat patients, therapeutic options remain without any significant changes. Currently, the best standard treatment is the maximum safe resection, followed by chemoradiation and adjuvant chemotherapy. We hope that new clinical trials with epigenetic target therapies could improve the responses to conventional treatments.

### 2.5. Epigenetic Modulation in Metastatic Melanoma

The main issue with metastatic melanoma lies in its chemoresistance. Currently, the new immunecheckpoint inhibitors against CTLA-4, PD-1 or PD-L1 have improved patient outcome. However, secondary genomic aberrations make tumor cells acquire rapid resistance to these therapies [176]. One of the risk factors associated with melanoma is UV radiation; this is due to changes in DNA methyltransferase and in histone acetylation, which leads to silencing of tumor-suppressive genes. In contrast, some dietary consumption of green tea and proanthocyanidins from grape seeds has the ability to block UV-induced epigenetic modification in the skin of *CIP1*/P21 or P16/*INK4a* [177]. The epigenetic modifications of melanoma are well defined; in fact, malignant transformation of peritumoral skin is due to epigenetic changes [178]. CC chemokine receptor 7 (*CCR7*) and CXC chemokine receptor 4 (*CXCR4*) are epigenetically upregulated in melanoma cells, and have the ability to induce metastasis of melanoma [179]. The following four tumor-suppressive genes are frequently hypermethylated in advanced melanoma: death-associated protein kinase (*DAPK*), O6-methylguanine DNA methyl-transferase (*MGMT*), *RAS* association domain family protein 1A (*RASSF1A*), and retinoic acid receptor-β2 (*RAR-β2*). The hypermethylation of *DAPK*, *MGMT* and *RASSF1A* is significantly lower in the early stages than in the advanced stages, whereas the incidence of hypermethylation of *RAR-β2* is highly similar in the early and advanced stages [180]. The HDAC inhibitor dacinostat (LAQ824) is able to restore retinoid sensitivity by reverting *RAR-β2* methylation in melanoma cells, and it achieved the highest benefits in combination with retinoids [181]. Also, TET proteins have been reported to play a crucial role in melanoma, since their ectopic expression of TET2 eradicates tumor proliferation and increases survival in vivo [37]. It has been described that the loss of histone acetylation and H3K4 (histone H3 Lysine 4) methylation in *BRAFV600E* and *PTEN* promote malignant transformation of melanocytes [182]. EZH2 is another factor expressed in metastatic melanoma; its depletion has been shown to restore P21/*CDKN1A* expression and arrest cell proliferation [183].

Concerning ncRNA, several studies have reported the importance of miRNA regulation in melanoma. For example, miRNA-125b is involved in the regulation of vitamin D receptor (VDR), and in the resistance of 1,25-dihydroxyvitamin D_3_, a potential therapy for metastatic melanoma [184]. Moreover, the expression of other miRNAs, from a large cluster of parentally imprinted regions located on chromosome 14q32, is significantly downregulated in melanoma, by epigenetic modulation. Interestingly, this miRNA cluster can be re-expressed with a combination of demethylating agents and histone deacetylase inhibitors. In this region, re-expression of mir-376a and mir-376c delayed cell growth and migration; moreover, one of the targets of both miRNAs is IGF1R, which is a tumor-prone factor in melanoma [185].

Since the largest clinical issue in the treatment of advanced melanoma patients is chemoresistance, the effort of researchers is centered around the discovery of a new treatment method to improve drug sensitivity. Interleukin-2 has exhibited potent antitumor activity in the fight against melanoma; nevertheless, its high toxicity has limited its use [186]. Treatment with SAHA is able to induce H3 and H4 hyperacetylation of P14/*ARF* promoter, and upregulate its expression [187]. Treatment with 5-aza-dC prevents the induction of DNMT1 and DNMT3b at the P16/*INK4A* promoter, leading to its subsequent activation [187]. Another treatment evaluated is allyl isothiocyanate (AITC), which has been reported to reduce cell proliferation and decrease the activation of HDACs, HATs, and other histone methyl transferases (HMTs). This approach is a very promising epigenetic therapy for advanced melanoma [188]. Some isothiocyanates, such as sulforaphane and iberin, could act over the epigenetic modulation of melanomas, and are currently under investigation [189]. Immune checkpoint-based therapy has improved patient lifespan from nine months to 2 years [190]. Perhaps, in the near future, the combination of anti-CTLA4 or anti-PD1 immune checkpoint inhibitors and epigenetic therapy could suppress the chemoresistance of metastatic melanoma [191].

Clinical trials with epigenetic therapy in metastatic melanoma have been mostly based on decitabine and other epigenetic modulating drugs, such as histone deacetylase inhibitors. A phase I clinical trial has explored the safety and tolerability of two epigenetic drugs, decitabine and panobinostat (a histone deacetylase inhibitor), in combination with temozolomide, to overcome chemoresistance in advanced melanoma (NCT00925132; Table 1). However, in this study, most of the patients exhibited disease progression [192]. Another clinical trial is testing the efficacy of oral azacitidine (CC-486) combined with pembrolizumab (NCT02816021; Table 1; Figure 1). Here, PD-1-naïve patients achieved a partial response (55% ORR), and accrual to this arm A continues; however, none of the patients with progression on prior PD-1 therapy, in arm B, have responded [193]. Other investigators have tested whether the action of vemurafenib (BRAF inhibitor) is more effective in combination with decitabine in low doses (NCT01876641; Table 1). Although the trial was terminated, due to a loss of funding, 3/14 patients achieved a complete response, 3/14 had a partial response, and 5/14 had stable disease. Moreover, its preclinical assessment demonstrated effectiveness of the combination, and a high potential in delaying chemoresistance [194]. Another clinical trial, performed in non-inflamed stage III/IV melanoma, is recruiting patients (NCT03765229; Table 1), and its clinical rationale is based on the induction of PD-L1 expression by the action of entinostat (HDAC inhibitor; Figure 2) [195]. The addition of anti-PD-1/anti-PD-L1 checkpoint inhibitors to HDAC inhibitors has been demonstrated to enhance the antitumor effect when compared to monotherapy, both in in vitro and in vivo models [196,197]. Another phase I clinical trial has evaluated the safety and efficacy of decitabine in combination with temozolomide (NCT00715793; Table 1). Here, there were 2/35 complete responses (CR), 4/35 partial responses (PR), 14/35 stable diseases (SD), 13/35 progressive diseases (PD), and the median overall survival was 12.4 months [198]. Another drug combination under investigation is tinostamustine with the anti-PD-L1 antibody nivolumab (NCT03903458; Table 1). Tinostamustine is an alkylating histone deacetylase inhibitor (HDACi), which resulted from the fusion of the alkylating agent bendamustine to the pan-HDACi vorinostat (Figure 2). This combination is expected to enhance the antineoplastic effect in refractory, locally advanced, or metastatic melanoma patients [199]. Also, the alkylating agent dacarbazine is the only drug approved by the Food and Drug Administration (FDA) as a therapy for advanced melanoma, with response rates between 7 and 13% [200].

Epigenetic therapies allow the reversibility of epigenetic modifications and are drawing attention to metastatic melanoma research, to prevent or delay the emergence of resistance to current standard treatments. Therefore, new discoveries in epigenetic therapies are expected to be evaluated in further clinical trials.

### 2.6. Epigenetic Modulation in Ovarian Cancer

Aggressive ovarian tumors (AOT) are the gynecological cancers with the highest mortality rate, probably because most AOT patients present advanced stages at diagnosis (stage III or IV), due to the lack of symptoms or unavailable specific screening biomarkers [201]. While response in the early stages is frequently acceptable, advanced tumors present a short progression driven by chemoresistance. Some translational research has shown that epigenetic aberrations are quite important in tumor initiation and development [202]. For example, the expression of HDAC2 hampers the DNA damage responses induced by platinum compounds, and contributes to the pathogenesis and chemoresistance of AOT [203]. In addition, the inhibition of H4K16 acetylation has been observed in AOT [204]. Further, hMOF, a member of the HATs family that acetylates H4K16, could also serve as an epigenetic biomarker for the diagnosis of malignant AOT, since patients with high expression levels of hMOF present improved survival when compared to those with low hMOF levels [205]. The presence of class I HDACs are able to induce the progression of AOT, and high expression of class I HDACs has also been detected in AOT patient samples. Furthermore, the expression of class I HDAC proteins has been considered a poor prognostic biomarker in AOT [206]. Cacan et al. have demonstrated that the downregulation of RGS2, an inhibitor of G-protein-coupled receptor proteins (GPCRs), confers chemoresistance of AOT cells, which is in part due to the repression of the promoter region of *RGS2* by class I HDACs [207]. Also, chemoresistance to platinum-based drugs has been associated with SIRT1 upregulation through the BRCA1–SIRT1–EGFR axis [208]. SIRT1 upregulation correlates to *TP53* inactivation by deacetylation [209]. SIRT3, in contrast, inhibits AOT cell migration via TWIST downregulation [210]. Other factors, such as EZH2, are overexpressed and have a direct positive correlation with AOT histological grade and tumor stage [211]. Further, 3-deazaneplanocin A (DZNEP) is a target for EZH2, with a promising anticancer efficacy against AOT [211]. Another EZH2 inhibitor, GSK126 (Figure 2), has demonstrated a better response in *ARID1A*-mutated patients [212]. Another study has associated LSD1 overexpression with AOT [213], and the combination of LSD1 with sodium butyrate increases most of the hallmarks of AOT [214,215]. Other factors, such as KDM3A, are crucial for AOT progression, undifferentiation, and platinum resistance, and have been identified as a potential target for AOT [216].

It is known that cancer modifies the microenvironment to inhibit the immune system. In this context, the overexpression of HLA-class I and II has been associated to AOT [217]. Epigenetically silenced hMLH1, together with cisplatin, could be an effective treatment, alongside decitabine and other HDAC inhibitors, such as belinostat (Figure 2), against AOT [218]. Chemoresistant tumor cells have inhibited the expression of OX-40L and 4-1BBL, two stimulator receptors of the immune system, with the concomitant overexpression of the immunosuppressive factor PD-L1 [219]. Indeed, HDAC1 and HDAC3 showed a strong association with OX-40L and 4-1BBL promoters, which contributes to OX-40L and 4-1BBL repression [219].

The inhibition of histone acetyltransferase is a new approach for the treatment of malignant AOT and its chemoresistance. The following three HDAC inhibitors have been approved by the FDA: romidepsin, panobinostat, and vorinostat (Figure 2). Trichostatin A (TSA), which exhibits a significant inhibition of class I and II HDACs, is able to activate P73 and trigger apoptosis in AOT cells [220]. Another study evaluated belinostat with carboplatin in platinum refractory AOT patients. However, the lack of drug activity concluded in the termination of the study [221]. Other authors initiated a phase Ib/II trial with recurrent AOT patients, to evaluate the clinical benefit of paclitaxel, carboplatin and belinostat [222]. Here, 3/35 patients presented a complete response, while 12/35 exhibited a partial response, with an ORR of 43%. It is remarkable that the median overall survival was not reached; thus, the results showed that paclitaxel + carboplatin + belinostat regimen demonstrated a clinical benefit. In a phase II study, vorinostat was evaluated for the treatment of recurrent AOT; however, vorinostat exhibited minimal activity as a single agent [223]. Another phase II trial evaluated the effect of hydralazine and magnesium valproate (NCT00404508; Table 1; Figure 1 and Figure 2). The clinical benefit with these epigenetic agents was observed in 80% of patients, which supported their use as epigenetic therapy to overcome chemoresistance in recurrent patients [224]. Another study tested decitabine as an epigenetic chemosensitizer to carboplatin plus a paclitaxel regimen (NCT02159820; Table 1). The study is supported by the fact that 5-aza-dC treatment is able to restore P27 expression and increases the sensitivity of tumor cells to cisplatin [225]. Another study aims to determine the optimal dose of oral azacitidine (CC-486) in combination with pembrolizumab, for the treatment of platinum-resistant or refractory AOT (NCT02900560; Table 1; Figure 1).

AOC is strongly influenced by epigenetic changes that affected DNA methylation and histone modifications. The first attempts to modify the epigenetic of AOC with drugs have achieved low response rates as single agents; thus, their combination with targeted therapies, based on the mutational burden of tumors, must be evaluated.

## 3. Conclusions

In the clinic, patients with highly aggressive tumors are presented with different prognoses, despite having a similar stage and grade of cancer. These observations could be explained by the tumor heterogeneity that is characterized by several epigenetic modification profiles [226]. Firstly, we must highlight several oncogenic point mutations associated with epigenetic regulators, such as *IDH1/2*, *EZH2* or *DNMT3A*. Moreover, not all mutations are tumor-prone, and we must consider tumor-suppressive factors such as KDM6A and CREBBP/P300 [227]. Finally, another important element is when DNA epigenetic modifications emerge with histone modifications, to inactivate the action of tumor-suppressive factors [228]. All these actions are crucial in the regulation of tumor initiation and development. Overall, these alterations could serve as molecular biomarkers to stratify high-risk patients into different groups and provide the best treatment strategy in each case. We are confident that all the positive results, obtained in hematopoietic malignancies in preclinical studies, provide a strong rationale for further trials in highly aggressive solid tumors, to improve patient survival and prevent chemoresistance. Most of the clinical trials with epigenetic drugs are in combination with standard chemotherapies; however, further research is needed with the combination of epigenetic drugs and targeted therapies.

## Figures and Tables

**Table 1:** Current clinical trials developed with epigenetic-based therapies in highly aggressive solid tumors in adulthood.

Identifier	Disease	Stage	Design	Drugs	Administration of ET	Epigenetic Target	Brief	Status
NCT02847000	Pancreatic cancer	Advanced	Early phase 1, single-arm, open-label, proof-of-concept clinical trial	**Decitabine**/tetra-hydrouridine	Orally	DNMT	Drug combination of decitabine and tetrahydrouridine in patients that have progressed through one or more lines of therapy. The most frequent adverse event was anemia and decitabine exhibited a limited systemic effect.	C
NCT01845805	Pancreatic cancer	Resected	Phase II trial, randomized, single group assignment, open label.	Oral **azacitidine** (CC-486)/nanoparticle albumin-bound paclitaxel or gemcitabine	Orally	DNMT	Azacitidine (CC-486) until recurrence, then first-line treatment: Abraxane or gemcitabine.	R
NCT04257448	Pancreatic cancer	Advanced	Open-label phase I/II study, non-randomized, sequential assignment, open label	**Romidepsin, azacitidine**, nab-paclitaxel, gemcitabine, durvalumab, lenalidomide	Subcutaneous	HDAC and DNMT	Azacitidine and/or romidepsin in combination with nab-paclitaxel/gemcitabine followed by sequential immune targeting with programmed death ligand (PD-L)1 blockade in combination with low-dose lenalidomide.	R
NCT02489903	SCLC, NSCLC, neuroendocrine tumors and ovarian epithelial cancer	Platinum refractory/resistant	Phase II study, randomized, parallel assignment, open label	**RRx-001**, cisplatin, etoposide, carboplatin, irinotecan, vinorelbine, Doxil, gemcitabine, taxane, Paclitaxel, nab-Paclitaxel, pemetrexed	Intravenously	DNMT	Participants with SCLC will receive one of the following: RRx-001 followed by platinum-doublet chemotherapy or platinum-based chemotherapy alone. Neuroendocrine, RRx-001 followed by platinum-doublet chemotherapy. NSCLC, RRx-001 followed by platinum-doublet chemotherapy. Participants with platinum refractory/resistant ovarian will receive one of the following: RRx-001 followed by platinum-doublet chemotherapy or chemotherapy alone.	A
NCT03901469	Triple-negative breast cancer	Without germline mutations of BRCA1 or BRCA2	Phase 2 study, non-randomized, single group assignment, open label	**ZEN-3694**, talazoparib	Orally	BET	Triple-negative breast cancer without germline mutations of BRCA1 or BRCA2	R
NCT01194908	Triple-negative breast cancer	Metastatic	Phase I/II trial, single group assignment, open label	**Decitabine, panobinostat**, tamoxifen	Intravenously	DNMT and HDAC	ER is silenced by methyl and histone groups. Reactivation of ER by demethylating inhibitors (such as decitabine) and histone deacetylase inhibitors (such as panobinostat) can remove these methyl and histone groups and reactivate ER with tamoxifen.	T
NCT01700569	Grade IV astrocytoma/glioblastoma	Complete or near-complete resection with unmethylated MGMT gene	A phase-1 dose-escalation study, single group assignment, open label,	**Folinic acid** concomitantly with temozolomide and radiation	Orally	DNMT	Temozolomide in combination with radiation therapy induces MGMT. Then, folinic Acid is able to lead MGMT methylation.	R
NCT00925132	Metastatic melanoma	Refractory/resistant to any prior treatment	Phase Ib/II trial with dose escalation, single group assignment, open label	Combination of temozolomide, **decitabine, panobinostat**	Orally	DNMT and HDAC	The treatment combination is proposed to unlock genes (Apaf-1) that may contribute to mechanisms that cause tumor growth. The triple agent was well tolerated.	T
NCT02816021	Metastatic melanoma	Unresectable stage III/IV metastatic melanoma	Phase II non-randomized, open label	**Oral azacitidine (CC-486)**, pembrolizumab	Orally	DNMT	The goal of this clinical research study is to learn if oral azacitidine (CC-486) and pembrolizumab (MK-3475) can help to control melanoma progression.	R
NCT01876641	Metastatic melanoma	BRAF-mutated tumors regardless of prior treatment	Phase 1/2 trial, single group assignment, open label	Vemurafenib, cobimetinib, **Decitabine**	Subcutaneous	DNMT	Improve the low therapy response rate with the combination of vemurafenib with decitabine plus cobimetinib.	T
NCT03765229	Metastatic melanoma	In non-Inflamed stage III/IV	An exploratory, open-label, single-arm, phase II study	**Entinostat**, pembrolizumab or any other PD-1/PD-L1 inhibitor	Orally	HDAC	Induction of epigenetic changes in tumor biology by entinostat to enhance treatment response, progression-free survival and incidence of adverse events.	R
NCT00715793	Metastatic melanoma	Unresectable stage IIIB/IV despite prior therapies	Single-arm phase I/II trial, single group assignment, open label	**Decitabine**, temozolomide	Intravenously	DNMT	The combination of decitabine and temozolomide may induce changes in DNA to improve clinical response. Determine the efficacy, safety and tolerability of the combination decitabine and temozolomide. This study obtained 18% ORR and 61% clinical benefit rate (CR + PR + SD)	C
NCT03903458	Metastatic melanoma	Refractory, locally advanced or metastatic	Open label, non-randomized, phase IB, single group assignment	**Tinostamustine**, nivolumab	N/A	HDAC	To assess the safety, tolerability and recommended dose of tinostamustine in combination with nivolumab and characterize potential predictive biomarkers of the combination treatment.	R
NCT00404508	Ovarian cancer and other solid tumors	Persistent or progression to first-line platinum-based chemotherapy	Randomized, double-blind phase II trial. Parallel assignment	Topotecan, **hydralazine, valproate**	Orally	DNMT and HDAC	Inhibitors of DNA methylation and HDAC inhibition may synergize the cytotoxicity of chemotherapy to improve response, progression-free survival and overall survival. A clinical benefit was observed in 80% patients and the main toxicity was hematologic.	C
NCT02159820	Ovarian cancer	Previously untreated	Open label, randomized, phase II to III, intergroup trial. Parallel assignment	**Decitabine**, paclitaxel, carboplatin	Intravenously	DNMT	Decitabine may trigger epigenetic reprogramming of tumor cells and possible immune cells could induce pronounced long-term clinical effect by chemosensitization and immunopotentiation.	R
NCT02900560	Ovarian cancer	Platinum-resistant/refractory	Open-label, non-randomized, four-cohort phase II. Parallel assignment	Pembrolizumab and **oral azacitidine (CC-486)**	Orally	DNMT	Four cohorts of combined oral azacitidine (CC-486) and intravenous pembrolizumab to evaluate the safety and efficacy. Mandatory tumor biopsies for DNA methylation analysis.	A

Drugs in **bold** are the epigenetic-based therapies. N/A: not available. ET: epigenetic therapy. DNMT: DNA methyltransferases. HDAC: histone deacetylases. BET: bromodomain and extra-terminal motif proteins. ORR: overall response rate. CR: complete response. PR: partial response. SD: stable disease. A: active, not recruiting. C: completed. R: recruiting. T: terminated.

## Data Availability

Not applicable.
